# Trends in HIV-2 Diagnoses and Use of the HIV-1/HIV-2 Differentiation Test — United States, 2010–2017

**DOI:** 10.15585/mmwr.mm6903a2

**Published:** 2020-01-24

**Authors:** Anne H. Peruski, Laura G. Wesolowski, Kevin P. Delaney, Pollyanna R. Chavez, S. Michele Owen, Timothy C. Granade, Vickie Sullivan, William M. Switzer, Xueyuan Dong, John T. Brooks, M. Patricia Joyce

**Affiliations:** ^1^Division of HIV/AIDS Prevention, National Center for HIV/AIDS, Viral Hepatitis, STD, and TB Prevention, CDC; ^2^ICF International, Inc., Atlanta, Georgia.

Since 2014, the recommended laboratory testing algorithm for diagnosing human immunodeficiency virus (HIV) infection has included a supplemental HIV-1/HIV-2 differentiation test to confirm infection type on the basis of the presence of type-specific antibodies (*1*). Correctly identifying HIV-1 and HIV-2 infections is vital because their epidemiology and clinical management differ. To describe the percentage of diagnoses for which an HIV-1/HIV-2 differentiation test result was reported and to categorize HIV type based on laboratory test results, 2010–2017 data from CDC’s National HIV Surveillance System (NHSS) were analyzed. During 2010–2017, a substantial increase in the number of HIV-1/HIV-2 differentiation test results were reported to NHSS, consistent with implementation of the HIV laboratory-based testing algorithm recommended in 2014. However, >99.9% of all HIV infections identified in the United States were categorized as HIV-1, and the number of HIV-2 diagnoses (mono-infection or dual-infection) remained extremely low (<0.03% of all HIV infections). In addition, the overall number of false positive HIV-2 test results produced by the HIV-1/HIV-2 differentiation increased. The diagnostic value of a confirmatory antibody differentiation test in a setting with sensitive and specific screening tests and few HIV-2 infections might be limited. Evaluation and consideration of other HIV tests approved by the Food and Drug Administration (FDA) that might increase efficiencies in the CDC and Association of Public Health Laboratories–recommended HIV testing algorithm are warranted.

Worldwide, the majority of HIV infections are HIV-1. HIV-2 occurs predominantly in West Africa, but has been reported in other countries, including the United States (*2*–*4*). When last assessed, 166 persons categorized as having HIV-2 infection were reported to CDC as cases of public health importance during 1987–2009 (*5*). NHSS is a case-based surveillance system for the United States (*6*); data include patient demographic characteristics, HIV transmission risk category, and laboratory test results. However, HIV infection type is not reported to or determined by NHSS. Consequently, CDC developed a surveillance definition for HIV-2 to determine the number of such cases and to describe the demographics of persons identified with the different HIV infection types in the United States.

For this analysis, the surveillance definitions for type of HIV infection include 1) HIV-2 mono-infection, defined as having an HIV-2-positive nucleic acid test (NAT) result or an HIV-2-positive HIV-1/HIV-2 differentiation test result and no evidence of HIV-1-RNA or DNA*; 2) HIV-1 and HIV-2 dual-infection, defined as having an HIV-2-positive or HIV-1-positive and HIV-2-positive antibody test result and positive HIV-1 and HIV-2 RNA or DNA test results; or 3) probable HIV-2 infection, defined as having an HIV-2-positive antibody test result (HIV-2 immunoassay or an HIV-1/HIV-2 antigen and antibody test) and no evidence of HIV-1 RNA or DNA. All remaining HIV diagnoses in NHSS were categorized as HIV-1. Data from NHSS were used to summarize patient demographics, HIV transmission risk category, and the number of pregnancies and perinatal transmissions according to HIV type. The estimated annual percentage change (*7*) was used to calculate the number of HIV diagnoses and the number of patients for whom an HIV-1/HIV-2 differentiation test result was reported to NHSS, both overall and for HIV-1 infections.

Laboratory test results were analyzed for persons with HIV infection diagnosed during 2010–2017 and reported to NHSS through December 2018. Two HIV-1/HIV-2 differentiation tests were available to U.S. laboratories during the analysis period: Multispot HIV-1/HIV-2 Rapid Test (Bio-Rad Laboratories), which was approved by FDA in 2004 and discontinued in 2016, and Geenius HIV-1/HIV-2 Supplemental Assay (Bio-Rad Laboratories), which was approved by FDA in 2014. However, in NHSS data, which of the HIV-1/HIV-2 differentiation tests was used cannot be determined.

During 2010–2017, of 327,700 diagnosed HIV infections in the United States, 327,502 (99.94%) were HIV-1. The remaining 198 (0.06%) diagnosed infections were classified as HIV-2 mono-infection (n = 102), dual HIV-1 and HIV-2 infection (n = 11), or probable but unconfirmed HIV-2 infections (n = 85) (Table 1). Demographic characteristics of persons with HIV-1 infection varied substantially from those with HIV-2 infection (including HIV-2, HIV-2 probable but unconfirmed, or dual HIV-1 and HIV-2 infection) (Table 2). Persons with HIV-2 infection were as likely to be female as male, were more frequently older, non-Hispanic black, had HIV infection attributed to heterosexual contact, had been born in countries where HIV-2 infection is endemic, and resided in the northeastern United States at the time of diagnosis. Among the 11 cases classified as dual HIV-1 and HIV-2 infection, six were among men, and five were among women. Eight persons were identified as having emigrated from a country where HIV-2 is endemic. Among the 99 women with confirmed or probable HIV-2 infection, nine had evidence of a pregnancy during or after diagnosis; however, no perinatal HIV-2 transmissions were reported to NHSS.

**TABLE 1:** Number of HIV diagnoses among persons aged ≥13 years, by diagnosis type — National HIV Surveillance System (NHSS), United States and six dependent areas,* 2010–2017

Diagnosis year	No.	Diagnosis type			
		HIV-1^†^	HIV-2^§^	HIV-2, probable, but unconfirmed^¶^	Dual HIV-1/HIV-2**
2010	44,086	44,066	7	13	0
2011	42,285	42,265	12	4	4
2012	41,467	41,443	9	12	3
2013	39,987	39,978	7	2	0
2014	40,667	40,635	22	9	1
2015	40,406	40,378	13	14	1
2016	40,121	40,085	18	18	0
2017	38,681	38,652	14	13	2
**Total**	**327,700**	**327,502**	**102**	**85**	**11**
**EAPC (95% CI)**		**–0.03 (–0.03 to –0.02)**	**12.0 (2.8 to 22.1)**	**11.4 (1.4 to 22.3)**	**–7.8 (–28.9 to 19.7)**

**Abbreviations:** CI = confidence interval; EAPC = estimated annual percentage change; HIV = human immunodeficiency virus.

* Data from CDC's NHSS collected through December 2018.

^†^ Diagnoses in NHSS with no evidence of an HIV-2, HIV-2 probable but unconfirmed, or dual HIV-1 and HIV-2 infections.

^§^ Diagnoses in NHSS with HIV-2 RNA or an HIV-2 positive differentiation test and no evidence of HIV-1 RNA or DNA.

^¶^ Diagnoses in NHSS with an HIV-2 positive antibody test and no evidence of HIV-1 RNA or DNA.

** Diagnoses in NHSS with HIV-1 and HIV-2 RNA or DNA.

**TABLE 2:** Characteristics of persons aged ≥13 years with diagnosed HIV infection — National HIV Surveillance System (NHSS), United States and six dependent areas,* 2010–2017

Characteristic	No. (%)	
	HIV-1^†^	HIV-2^§^
**Total**	**327,502 (100)**	**198 (100)**
**Age group (yrs)**		
13–24	71,893 (22)	20 (10.1)
25–34	100,937 (30.8)	30 (15.2)
35–44	67,462 (20.6)	35 (17.7)
45–54	55,957 (17.1)	46 (23.2)
≥55	31,253 (9.5)	67 (33.8)
**Sex**		
Male	262,520 (80.2)	99 (50.0)
Female	64,982 (19.8)	99 (50.0)
**Race/Ethnicity**		
Black, non-Hispanic	141,712 (43.3)	147 (74.2)
White, non-Hispanic	84,848 (25.9)	15 (7.6)
Hispanic	80,291 (24.5)	21 (10.6)
Other	20,651 (6.3)	15 (7.6)
**Transmission category**		
Male-to-male sexual contact	210,250 (64.2)	50 (25.3)
Heterosexual contact	84,063 (25.7)	121 (61.1)
Injection-drug use (IDU)	20,966 (6.4)	23 (11.6)
Male-to-male sexual contact/IDU	11,613 (3.5)	2 (1.0)
Other	610 (0.2)	2 (1.0)
**Birth country**		
United States	205,370 (62.7)	44 (22.2)
Other countries	70,647 (21.6)	37 (18.7)
Unknown	48,222 (14.7)	28 (14.1)
Countries where HIV-2 is endemic^¶^	3,263 (1)	89 (44.9)
**U.S. Census region of residence at diagnosis**		
South	163,204 (49.8)	62 (31.3)
West	61,380 (18.7)	16 (8.1)
Northeast	55,593 (17)	109 (55.1)
Midwest	42,069 (12.8)	11 (5.6)
U.S. dependent areas	5,256 (1.6)	0 (—)

**Abbreviation:** HIV = human immunodeficiency virus.

* Data from CDC's NHSS collected through December 2018.

^†^ Diagnoses in NHSS with no evidence of HIV-2, HIV-2 probable but not confirmed, or dual HIV-1 and HIV-2 infections.

^§^ Diagnoses in NHSS of HIV-2, HIV-2 probable but not confirmed, or dual HIV-1 and HIV-2 infections.

^¶^ Angola, Benin, Burkina Faso, Cape Verde, Côte d’Ivoire, the Gambia, Ghana, Guinea, Guinea-Bissau, Liberia, Mali, Mauritania, Mozambique, Niger, Nigeria, São Tomé, Senegal, Sierra Leone, and Togo.

The number of persons with HIV infection whose report to NHSS included an HIV-1/HIV-2 differentiation test result increased by an estimated 21.2% per year (95% confidence interval [CI] = 21.0–21.4) during 2010–2017 (Table 3). Concurrently, the number of confirmed and probable HIV-2 infections increased by an estimated 12.0% per year (95% CI = 2.8–22.1) and 11.4% per year (95% CI = 1.4–22.3), respectively, during 2010–2017 (Table 1). Although the number of HIV-1/HIV-2 differentiation test results continued to increase during 2014–2017 by an estimated 6.4% per year (95% CI = 6.2%–6.9%), the number of persons with confirmed or probable HIV-2 infections did not change with an estimated annual percentage change including zero, –9.5% per year (95% CI = –27.1% to 12.3%) and 14.3% per year (95% CI = –10.1% to 45.4%), respectively.^†^ Among persons with confirmed HIV-1 infection, 356 included false-positive HIV-2 results from an HIV-1/HIV-2 differentiation test (Table 3), and the number of false positive reports increased an estimated 18.8% per year (95% CI = 13.3–24.5) relative to all HIV diagnoses. However, the number of false positive reports relative to those whose report to NHSS included an HIV-1/HIV-2 differentiation test decreased during the study period by 6.2% (95% CI = –10.7% to –1.5%) (Table 3).

**TABLE 3:** HIV differentiation testing for persons aged ≥13 years with diagnosed HIV infection — National HIV Surveillance System (NHSS), United States and six dependent areas,* 2010–2017

Diagnosis year	No. of HIV diagnoses	No. (%)		
		Overall persons tested with an HIV-1/HIV-2 differentiation test	Persons with diagnosed HIV-1,^†^ tested with an HIV-1/HIV-2 differentiation test	Persons with diagnosed HIV-1,^†^ tested with an HIV-1/HIV-2 differentiation test that was falsely positive for HIV-2^§^
2010	44,086	8,761 (19.9)	8,755 (19.9)	26 (0.3)
2011	42,285	8,865 (21.0)	8,850 (20.9)	23 (0.3)
2012	41,467	9,997 (24.1)	9,987 (24.1)	19 (0.3)
2013	39,987	14,105 (35.3)	14,099 (35.3)	25 (0.2)
2014	40,667	26,147 (64.3)	26,120 (64.3)	68 (0.3)
2015	40,406	31,576 (78.2)	31,551 (78.1)	87 (0.3)
2016	40,121	32,346 (80.6)	32,313 (80.6)	65 (0.2)
2017	38,681	31,458 (81.3)	31,432 (81.3)	43 (0.1)
**Total**	**327,700**	**163,255 (49.8)**	**163,107 (49.8)**	**356 (0.2)**
**EAPC (95% CI)**		**21.2 (21.0 to 21.4)**	**21.2 (21.1 to 21.4)**	**–6.2 (–10.7 to –1.5)**

**Abbreviations:** CI = confidence interval; EAPC = estimated annual percentage change; HIV = human immunodeficiency virus.

* Data from CDC’s NHSS collected through December 2018.

^†^ Diagnoses in NHSS with no evidence of an HIV-2, HIV-2 probable but unconfirmed, or dual HIV-1 and HIV-2 infection.

^§^ Percentage of those who ever received an HIV-1/HIV-2 differentiation test.

## Discussion

These results are consistent with the previously reported findings from 1987–2009 that HIV-2 remains a rare diagnosis in the United States (*5*). Use of the HIV-1/HIV-2 differentiation test increased steadily throughout the study period, although after 2014 the number of confirmed or probable HIV-2 infections remained stable. The number of persons with confirmed HIV-1 infection who had a false-positive HIV-2 test result by using the HIV-1/HIV-2 differentiation test was greater than the total number of confirmed and probable HIV-2 diagnoses combined. In these cases, HIV antibody cross-reactivity likely caused the false-positive reaction and necessitated additional time and testing to resolve (*8*).

Although HIV-2 is rare, correct diagnosis is vital for ensuring correct clinical management. Persons with HIV-2 who have an incorrect HIV-1 diagnosis and are treated with nonnucleoside reverse-transcriptase inhibitors, to which HIV-2 is intrinsically resistant, might fail to suppress an HIV-2 viral load (*9*). Without commercially available HIV-2 viral load tests, HIV-2 infection might not be recognized, or might require additional testing to determine HIV status. This step might include having to send specimens to specialized laboratories that perform a laboratory-developed HIV-2 NAT.

The findings in this report are subject to at least three limitations. First, the definition used to define HIV-2 infection using test results entered into the Enhanced HIV/AIDS Reporting System,^§^ (an application for collecting, storing, and retrieving HIV-related data), was developed for use with surveillance data to report epidemiologic trends. Identification of type of HIV diagnosis for the management of patients might require additional diagnostic tests that are beyond the scope of this study. Second, for 61 (33%) HIV-2 diagnoses (probable and confirmed) with missing HIV-1 NAT results, the possibility of HIV-2 infection or identification of dual infection could not be ruled out. An HIV-2 NAT might also have helped confirm these infections, but no FDA-approved commercially available test exists. Moreover, HIV-2 infection results in lower levels of circulating virus compared with those of HIV-1 infection. Finally, evidence of pregnancy in women with HIV infection is underreported to NHSS (*10*). Although this can result in an underestimation of the number of pregnant women with HIV-2, reporting of perinatal HIV infection is robust, thus increasing the likelihood that perinatal HIV-2 infection would have been recognized.

Despite increasing use of the HIV-1/HIV-2 differentiation test, few HIV-2 infections are diagnosed in the United States. CDC continues to recommend that laboratories follow the laboratory-based algorithm with the HIV-1/HIV-2 differentiation test as the second step. Use of an HIV-1 NAT in the algorithm would likely distinguish type of HIV infection for the majority of diagnoses in the United States. Follow-up testing of specimens that remain ambiguous regarding HIV type after testing with an HIV-1 NAT is also recommended.^¶^However, updates to the laboratory-based testing algorithm merit consideration in the United States. This could include development of new FDA-approved tests to reduce the time to HIV diagnosis and treatment, primarily for HIV-1, but in rare cases, for HIV-2.

**Summary**

**What is already known about this topic?**

Since 2014, CDC has recommended using an antibody-based HIV-1/HIV-2 differentiation test as part of a laboratory-based algorithm to confirm HIV-1 and HIV-2 infections.

**What is added by this report?**

During 2010–2017, use of the HIV-1/HIV-2 differentiation test increased, but the number of confirmed HIV-2 diagnoses remained <0.1%. In addition, the overall number of false positive HIV-2 test results produced by the HIV-1/HIV-2 differentiation increased.

**What are the implications for public health practice?**

CDC recommends that laboratories continue to follow the laboratory-based algorithm with the HIV-1/HIV-2 differentiation test as the second step. However, updates to the laboratory-based testing algorithm merit consideration in the United States where HIV-2 infections remain rare.

## Footnotes

* A positive quantitative or qualitative nucleic acid test result or molecular sequence data for an HIV-1 genotypic drug-resistance test.

† 2014–2017 total HIV diagnoses = 159,875.

§ https://doi.org/10.3886/ICPSR34725.v1.

¶ https://stacks.cdc.gov/view/cdc/50872.
